# Clozapine-induced myocarditis: electronic health register analysis of incidence, timing, clinical markers and diagnostic accuracy

**DOI:** 10.1192/bjp.2021.58

**Published:** 2021-12

**Authors:** Aviv Segev, Ehtesham Iqbal, Theresa A. McDonagh, Cecilia Casetta, Ebenezer Oloyede, Susan Piper, Carla M. Plymen, James H. MacCabe

**Affiliations:** Department of Psychosis Studies, Institute of Psychiatry, Psychology and Neuroscience, King's College London, UK; Shalvata Mental Health Centre, Israel; and Sackler Faculty of Medicine, Tel Aviv University, Israel; The Department of Biostatistics and Health Informatics, Institute of Psychiatry, Psychology and Neuroscience, King's College London, UK; Cardiology Department, King's College Hospital and King's College London, UK; Department of Psychosis Studies, Institute of Psychiatry, Psychology and Neuroscience, King's College London, UK; and National Psychosis Service, South London and Maudsley NHS Foundation Trust, UK; Department of Psychosis Studies, Institute of Psychiatry, Psychology and Neuroscience, King's College London, UK; and Pharmacy Department, South London and Maudsley NHS Foundation Trust, UK; Cardiology Department, Hammersmith Hospital, Imperial College Healthcare NHS Trust, UK

**Keywords:** Antipsychotics, drug interactions and side-effects, psychotic disorders, schizophrenia, risk assessment

## Abstract

**Background:**

Clozapine is associated with increased risk of myocarditis. However, many common side-effects of clozapine overlap with the clinical manifestations of myocarditis. As a result, there is uncertainty about which signs, symptoms and investigations are important in distinguishing myocarditis from benign adverse effects of clozapine. Clarity on this issue is important, since missing a diagnosis of myocarditis or discontinuing clozapine unnecessarily may both have devastating consequences.

**Aims:**

To examine the clinical characteristics of clozapine-induced myocarditis and to identify which signs and symptoms distinguish true myocarditis from other clozapine adverse effects.

**Method:**

A retrospective analysis of the record database for 247 621 patients was performed. A natural language processing algorithm identified the instances of patients in which myocarditis was suspected. The anonymised case notes for the patients of each suspected instance were then manually examined, and those whose instances were ambiguous were referred for an independent assessment by up to three cardiologists. Patients with suspected instances were classified as having confirmed myocarditis, myocarditis ruled out or undetermined.

**Results:**

Of 254 instances in 228 patients with suspected myocarditis, 11.4% (*n* = 29 instances) were confirmed as probable myocarditis. Troponin and C-reactive protein (CRP) had excellent diagnostic value (area under the curve 0.975 and 0.896, respectively), whereas tachycardia was of little diagnostic value. All confirmed instances occurred within 42 days of clozapine initiation.

**Conclusions:**

Suspicion of myocarditis can lead to unnecessary discontinuation of clozapine. The ‘critical period’ for myocarditis emergence is the first 6 weeks, and clinical signs including tachycardia are of low specificity. Elevated CRP and troponin are the best markers for the need for further evaluation.

## Background

Clozapine is the ‘gold-standard’ drug in the management of treatment-resistant schizophrenia (TRS)^1,2^ defined as inadequate response to at least two trials of antipsychotic medication of adequate dose and duration.^3^ Despite its proven and widely accepted clinical benefits, clozapine use has been limited by potentially life-threatening side-effects, especially haematological, metabolic and cardiac effects.^4^ One of the most significant adverse effects of clozapine is myocarditis.

Clozapine-induced myocarditis (CIM) is clinically and pathologically defined as inflammation of the myocardium as a result of clozapine administration. Although the pathogenesis of CIM is not entirely clear, an immune-mediated mechanism responsible for the inflammation of the myocardium and pericardium^5^ has been postulated, given its early onset in clozapine treatment, and the presence of eosinophilic infiltrates in myocardium.^6^ A direct selective cardiotoxic effect of clozapine metabolites owing to altered metabolism and oxidative stress has also been hypothesised.^7^

Although the risk of potentially fatal myocarditis with clozapine is estimated to be as low as 0.015–0.188%.^8^ Most guidelines recommend vigilance for signs and symptoms of myocarditis during clozapine use, especially during the first weeks of treatment. However, the clinical features of myocarditis are non-specific and highly variable. The presentation can range from electrocardiogram (ECG) abnormalities in asymptomatic patients, subtle signs and symptoms of heart failure such as fatigue, to a severe clinical picture of heart failure with shortness of breath and chest pain, arrhythmia and sudden cardiac death.^9,10^ Some patients may not even describe any symptoms or present with any signs.

The variability of clinical presentation and frequently insidious onset of symptoms make the true incidence and prevalence of CIM difficult to determine,^11^ and benign cases of CIM may go undetected and remit without the need to stop clozapine treatment.^12^ Current studies report a range between 0.2% and 3.4%,^5,13^ with distinguishably higher rates reported by Australian studies. Some authors have attributed this variation in incidence rates to better monitoring and case identification in Australia.^12^ As preventative measures, current guidelines suggest a gradual dose titration, serial ECG and a high level of suspicion for non-specific symptoms of myocarditis (such as malaise, chest pain, palpitation).^14^ Avoiding concomitant valproate use has also been proposed to reduce the risk of developing CIM.^15^

## Aims

Despite being first described over 20 years ago,^12,16,17^ current knowledge on the management of CIM has been predominantly limited to case reports. As such, there is a lack of consensus on managing suspected CIM across published guidelines.^18^ In addition, in the context of suspected CIM, the risk–benefit analysis of re-challenging patient on clozapine is very difficult given the paucity of evidence.^7,19^ The aim of this study was to assess the incidence and clinical characteristics of CIM in a large comprehensive electronic health record database.

## Method

### Sample and setting

The cohort was derived from the South London and Maudsley (SLaM) NHS Foundation Trust Biomedical Research Centre Case Register. SLaM is a National Health Service Foundation Trust, one of the largest mental health organisations in Europe, responsible for the psychiatric care of over 1.3 million residents in South London. The electronic health records of SLaM patients are extracted and extensively de-identified, forming a comprehensive, anonymised clinical database, named the Clinical Research Interactive Search (CRIS), fully described elsewhere.^20^ For this study, we have included all patients registered in CRIS, having more than one entry in their case records (as a single-entry record would disrupt the algorithm). Patient demographics and clinical data were obtained from both structured fields and free-text fields in the CRIS medical records. The data was collected from all notes recorded in CRIS until February 2019.

### Identification of suspected myocarditis

A natural language processing (NLP) algorithm designed to identify adverse drug events was used to scan the entire CRIS database to retrieve potential instances of suspected myocarditis (regardless of clozapine use). The NLP application works by identifying related words and then assigning each reference as positive or negative according to the context surrounding the keyword.^21^ The algorithm identified patients with potential suspected myocarditis if the case notes included at least two positive references of ‘myocarditis’. These criteria were used as it seemed unlikely that a true suspicion of myocarditis would not be documented more than once in any of the clinical notes, discharge letters, referrals and summaries. Anonymised case records were then examined manually by a psychiatrist (A.S.) to ascertain whether myocarditis was indeed suspected. ‘Suspected myocarditis’ was defined when a suspicion was recorded and any type of clinical action was taken that indicated a suspicion of myocarditis (i.e. increasing frequency of vital signs monitoring, ordering an ECG or lab workup, consulting with a cardiologist, discussing cessation and referring to an accident and emergency department to rule out or diagnose myocarditis). To avoid lack of adequate clinical information, episodes of myocarditis that has occurred before presentation to SLaM services were excluded.

### Myocarditis definition

Instances of suspected myocarditis were classified into three groups: (a) confirmed myocarditis, (b) Myocarditis ruled out, and (c) undetermined. Endomyocardial biopsy is the gold standard for diagnosing myocarditis, but this procedure is rarely performed or indicated in clinical practice owing to its significant invasiveness and low diagnostic yield. In lieu of this, echocardiography and in particular cardiac magnetic resonance imaging (MRI) are considered the best non-invasive imaging modalities to aid diagnosis. However, these investigations may not be available in a timely fashion to clinicians. Moreover, premature or delayed echocardiograms may not detect the expected anomaly of systolic dysfunction to diagnose myocarditis. Although cardiac MRI is superior in its ability to identify the myocardial inflammation regardless of structural abnormalities, it may be challenging for psychiatric patients who are agitated. Previous studies have thus used an alternative set of criteria to establish a diagnosis of myocarditis,^13^ which enable the use of a combination of clinical symptoms (such as chest pain), signs (such as fever), lab results (such as troponin or creatine kinase (CK)) and ECG instead of the gold-standard test.

In the current study, myocarditis status was determined in two steps. First, instances where myocarditis could confidently be confirmed or refuted were identified. The criteria for this step were devised by the study cardiologists (T.A.M., S.P.) and instances were assigned to the three groups: confirmed myocarditis, myocarditis ruled out and ‘undetermined’ as follows.

#### Confirmed myocarditis

- Troponin, echocardiogram/cardiac magnetic resonance (cMR) and documented cardiological opinion all support the diagnosis, or
- positive echocardiogram/cMR and positive troponin test, while cardiology opinion not done or not documented, or
- positive echocardiogram/cMR and supporting cardiology opinion, while troponin test not done or not documented.

#### Myocarditis ruled out

- Troponin, echocardiogram/cMR and documented cardiological opinion all rejecting the diagnosis, or
- two of the criteria in (a) refuting the diagnosis, with the third not done or not documented, or
- one or none of the criteria in (a) refuting the diagnosis, with the other two or three not done or not documented, as well as: all other tests (ECG, CK, C-reactive protein (CRP), full blood count) are not suggestive of myocarditis, symptomatology non-specific (dyspnoea, palpitation, chest pain, shock signs) and either established alternative diagnosis or clinical team stopped further workup as myocarditis seemed unlikely (minimal severity, self-resolution) without cessation of clozapine.

#### Myocarditis undetermined

- All instances not assigned to either of the two former definitions.

The instances that were ‘undetermined’ by these criteria, were sent to two independent cardiologists (T.A.M., S.P.), who reviewed the cases using all information available from the anonymised health record, and according to the European Society of Cardiology diagnostic criteria for myocarditis.^22^ instances were re-assigned to either ‘confirmed myocarditis’ or ‘myocarditis ruled out’ groups. instances that could not be confidently confirmed or ruled out as myocarditis following cardiology review remained in the third group of ‘undetermined’. Each cardiologist was masked to the other's decision. Where the two cardiologists did not agree, the details were sent to a third cardiologist (C.M.P., masked to the previous rulings) to aid in arbitration.

The troponin test was used to refute the diagnosis only if undertaken within 48 h of the suspicion raised.

### Medication data

The electronic health record of each patient who had a suspected event of myocarditis was reviewed to determine the concurrent medication regime, including antipsychotics, all other psychiatric and non-psychiatric medications, as well as the recorded dose. Clozapine initiation, if relevant, was defined as the first day of clozapine administration. If a patient had more than one course of clozapine, the most recent trial prior to the suspected myocarditis was considered.

### Statistical analysis

A receiver operating characteristic (ROC) curve analysis was performed to evaluate the clinical significance of related clinical measures. The interrater reliability was assessed using Cohen's kappa coefficient. Statistical analysis was performed using SPSS version 25.

### Ethical approval

Ethical approval for the use of CRIS as a research data-set was given by Oxfordshire Research Ethics Committee C (08/H0606/71) and the CRIS oversight committee granted permission for this study.

## Results

**Fig. 1:**
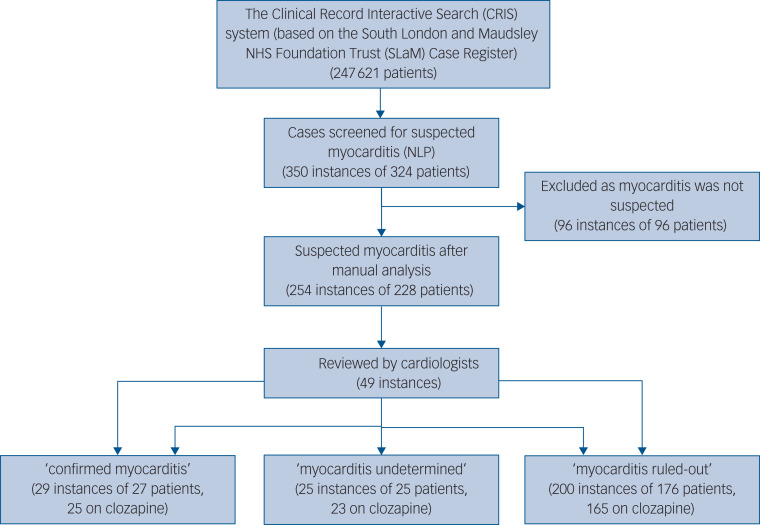
Selection of study instances and study procedures. NLP, natural language processing.

Data were available for 247 621 SLaM patients registered at the SLaM database between 2007 and February 2019. As illustrated in Fig. 1, 350 instances of suspected myocarditis were identified by the NLP application, arising from 324 patients, as 24 patients had more than one instance of suspected myocarditis.

The average number of positive mentions of myocarditis among the patients identified was 6.29 (s.d. = 10.11, minimum 2, maximum 127). After manual analysis, suspected myocarditis was identified in 254 instances, from 228 patients. In the remaining 96 instances, the references to myocarditis were mainly in the context of historical events (prior to contact with mental health services), describing family history, mentioned as a possible side-effect or with a low level of suspicion that did not prompt any clinical action. Of the 254 instances, 241 (94.9%) were prescribed clozapine at the time of the event, 5 (2.0%) were prescribed other antipsychotics and 8 (3.1%) were not taking antipsychotics at the time of the mentioned myocarditis.

In total 49 (19.3%) instances were deemed as ‘myocarditis undetermined’ per the first step of classification (see Methods section above), and therefore referred to the cardiologists for review. Cardiologists’ agreement was very good (85.7%) and kappa score was 0.79, indicating substantial agreement. In all the seven instances (14.3%) where cardiologists differed in opinion, the discrepancy included the intermediate category (‘undetermined’). Thus, there were no instances where one cardiologist classified an instance as ‘probable’ myocarditis while the other ruled the diagnosis out. In all instances where arbitration was needed, the third cardiologist agreed with one of the two previous cardiologists.

After the two-step classification process, 29 of the 254 suspected instances (11.4%) were identified as myocarditis and 25 (9.8%) instances had insufficient information to determine myocarditis. Myocarditis was ruled out in the remaining 200 (78.7%).

Of the 29 instances confirmed to be myocarditis, attributed to 27 patients, 25 patients were prescribed clozapine at the time of myocarditis, and 2 were not prescribed any antipsychotics. Of the 25 instances classified as probable myocarditis, attributed to 25 patients, 23 were prescribed clozapine and 2 were not on any antipsychotics.

The symptoms, signs and lab results of the myocarditis-confirmed and ruled-out groups are presented in Table 1. In addition, this table presents the prevalence of previously suggested cut-offs for clozapine cessation because of myocarditis suspicion: CRP increase over 100 mg/L and troponin increase over twice the normal limit.^14^

**Table 1:** Demographics, symptoms, signs and lab results by diagnostic group (for cloazpine administered instances)

	Myocarditis confirmed (*n* = 27)	Myocarditis ruled out (*n* = 191)	Myocarditis undetermined (*n* = 23)
*Demographic characteristics*			
Gender, *n* (%) male	23 (85.2)	137 (71.7)	15 (65.2)
Ethnicity, %			
White	12 (44.4)	37.9	10 (43.5)
Black	12 (44.4)	47.9	11 (47.8)
Other	3 (11.1)	14.2	2 (8.7)
Age, years: mean (%)	36.87 (13.55)	38.0 (12.54)	35.13 (13.37)
Age bands, years: *n* (%)			
0–29	11 (40.7)	63 (33.0)	10 (43.5)
30–39	4 (14.8)	45 (23.6)	7 (30.4)
40–49	6 (22.2)	49 (25.7)	3 (13.0)
50–80	6 (22.2)	34 (17.8)	3 (13.0)
*Clinical characteristics*			
Symptoms, *n* (%)			
Chest pain	12 (44.4)	34 (18.4)	6 (40.0)
Malaise	13 (56.5)	39 (21.4)	8 (57.1)
SOB	8 (34.8)	27 (14.6)	1 (7.1)
Oedema	0 (0.0)	1 (0.5)	0 (0.0)
Palpitations	1 (4.5)	8 (4.3)	2 (14.3)
*Vital signs^a^*			
Mean temperature, mean (s.d.)	37.96 (1.12)	37.49 (1.01)	38.10 (1.61)
Abnormal temperature, *n* (%)	13 (59.1)	59 (44.7)	5 (62.5)
Mean blood pressure, mean (s.d.)	115.3/70.2 (19.0/14.1)	126.1/82.2 (18.4/11.4)	131.6/85.3 (27.6/13.8)
Abnormal blood pressure, *n* (%)	6 (30.0)	37 (25.9)	3 (30.0)
Mean % saturation, mean (s.d.)	96.50 (1.87)	97.56 (5.43)	95.67 (5.82)
Abnormal saturation, *n* (%)	1 (7.1)	8 (7.8)	1 (16.7)
Mean pulse rate, mean (s.d.)	116.30 (11.86)	112.25 (14.30)	114.67 (12.85)
Abnormal pulse rate, *n* (%)	23 (100.0)	156 (89.7)	12 (100.0)
*Lab*^b^			
Mean CRP, mean (s.d.)	87.39 (64.27)	18.80 (27.89)	86.76 (62.41)
Abnormal CRP (>5 mg/L), *n* (%)	18 (100.0)	68 (53.1)	5 (55.6)
Mean CK, mean (s.d.)	169.92 (112.50)	575.98 (1779.46)	167.57 (193.36)
Abnormal CK (>150 Units/L), *n* (%)	7 (58.3)	50 (60.2)	1 (14.3)
Mean troponin mg/L, mean (s.d.)	1926.54 (4501.22)	18.33 (69.18)	121.42 (212.21)
Abnormal troponin (>16 ng/L), *n* (%)	24 (100.0)	14 (10.4)	11 (91.7)
Mean WBC, mean (s.d.)	9.36 (2.63)	8.42 (3.51)	11.58 (4.03)
Abnormal WBC (>11 × 10^9^/L), *n* (%)	6 (28.6)	25 (18.4)	5 (55.6)
Mean eosinophils, mean (s.d.)	0.87 (1.37)	0.37 (0.85)	0.28 (0.14)
Abnormal eosinophils (>0.4× 10^9^/L), *n* (%)	10 (62.5)	16 (13.6)	1 (16.7)
Any ECG anomalies, *n* (%)	10 (47.6)	26 (15.7)	3 (23.1)

SOB, Shortness Of Breath. CRP, C-reactive protein; CK, creatine kinase; WBC, white blood count; ECG, electrocardiogram.

a. At the time of first suspicion.

b. First measure to be taken upon raising the suspicion, or as triggers for suspicion.

**Fig. 2:**
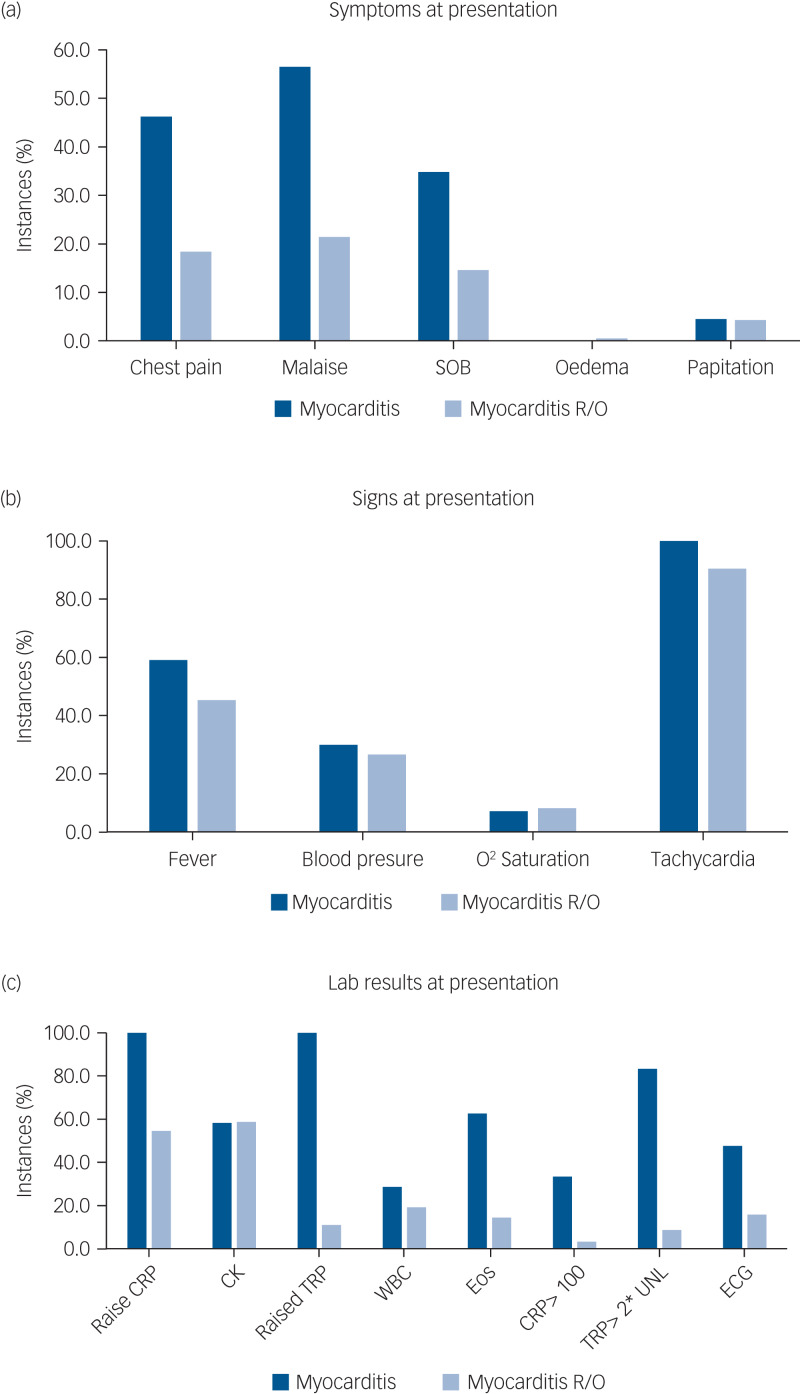
Prevalence of (a) symptoms, (b) positive signs and (c) lab results at suspected instances of myocarditis according to final diagnosis. CRP, C-reactive protein; CK, creatinine kinase; TRP, troponin; WBC, white blood count; Eos, eosinophils; ECG, electrocardiogram; R/O, ruled out; UNL, upper normal limit. Cut-off used: fever >37.5; abnormal blood pressure systolic >140 or <100, or diastolic >90 or <70; saturation <95%, tachycardia heart rate >90, CRP >6 mg/L, CK >150 U/L, troponin >16 ng/dL, WBC >11 000/mL, eosinophils >400/mL.

The symptoms, signs and lab results were tested for their sensitivity and specificity. Although almost all measures were more prevalent in the myocarditis-confirmed group (Fig. 2), most were not sensitive and not specific. Three measures showed very high sensitivity of 100% – tachycardia, elevated CRP and positive troponin. All others fell below 65% sensitivity. However, from the three sensitive markers, only troponin level above the cut-off level had high specificity (89.1%), whereas tachycardia and CRP were non-specific (9.5% and 45.5%, respectively). The positive predictive values for troponin, CRP and tachycardia were 50.0%, 29.9% and 12.3%, respectively, and the negative predictive value for all three was 100%.

**Fig. 3:**
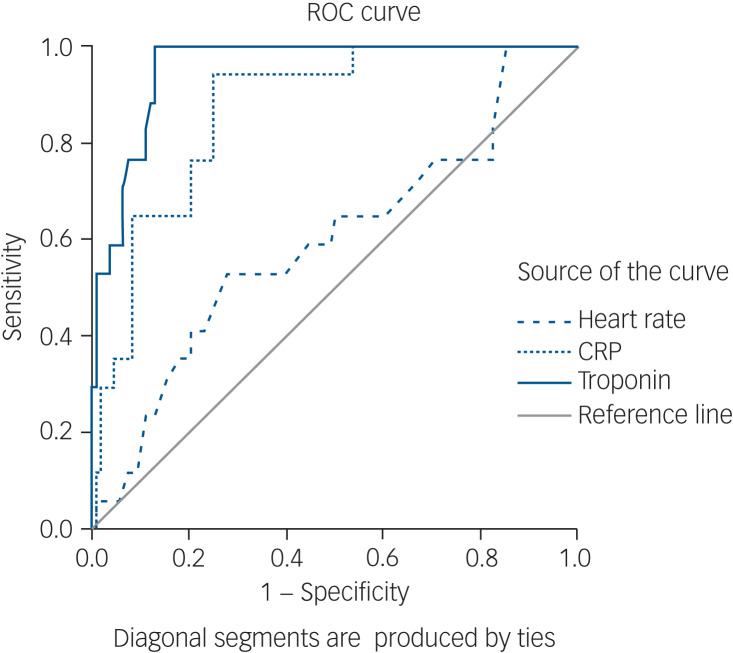
Receiver operating characteristic (ROC) curve of troponin, C-reactive protein (CRP) and heart rate.

Further analysis of these three measures as continuous measures using a ROC curve analysis (Fig. 3) showed that both troponin and CRP had very high area under the curve (AUC) values (0.975 and 0.896, respectively), whereas heart rate had a poorer AUC of 0.597. The two measures previously suggested in the literature, CRP over 100 and troponin over twice the normal limit, differed in their diagnostical properties: although CRP > 100 lacked sensitivity (33.3%), it had high specificity (96.7%). Troponin over twice the normal limit was sensitive (83.3%) and maintained high specificity (91.4%).

**Fig. 4:**
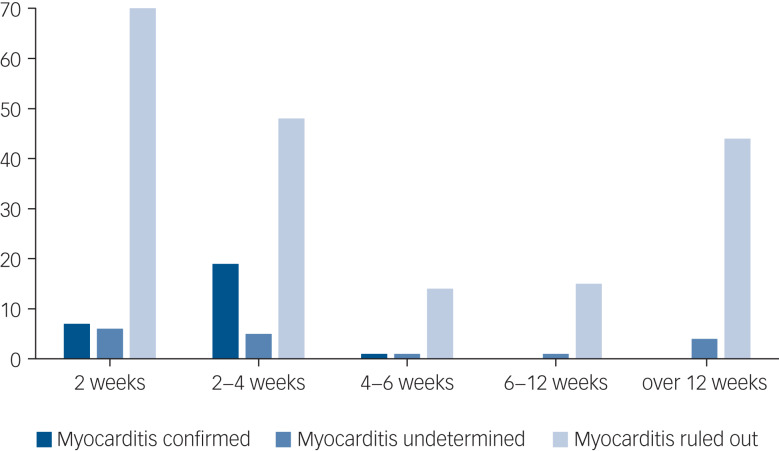
Period prevalence of suspected myocarditis instances, divided by final diagnosis.

The time from clozapine initiation to diagnosis of myocarditis was 2–42 days (mean 17.37, s.d. = 7.41, Fig. 4). Of the 27 patients prescribed clozapine, 22 were on their first clozapine trial at the time of myocarditis, and 5 had prior use of clozapine. Of these five patients, three had previously stopped clozapine because of suspected myocarditis, one because of chest pain and suspected chest infection and one because of neutropenia. The initial trial of clozapine for these five re-challenged patients was stopped within 45 days of initiation because of the described adverse events. The mean clozapine dose at the time of myocarditis emergence was 199.5 mg (25–300 mg, s.d. = 93.3). None of these instances occurred while undergoing rapid titration, and one occurred during the slow titration protocol. Only 1 patient (out of the 27 clozapine users) was administered clozapine only, the others were also prescribed valproate (*n* = 6), lithium (*n* = 3), benzodiazepines (*n* = 7), selective serotonin reuptake inhibitors (*n* = 4), additional antipsychotic (*n* = 6: olanzapine *n* = 2, quetiapine *n* = 2, amisulpride *n* = 1, haloperidol *n* = 1), mirtazapine (*n* = 1), metformin (*n* = 4) and hyoscine (*n* = 5). For six patients, data on concomitant medication was not available.

Clozapine was stopped in 35 (18.3%) of instances where myocarditis was suspected but eventually ruled out, and 88.9% (*n* = 24) stopped clozapine treatment in the confirmed myocarditis group. In the remaining 11.1% (*n* = 3) where the criteria for myocarditis were met but the clozapine was continued, the medical notes did not indicate any cardiac sequalae during the period in which clozapine was maintained, nor after it was stopped, and none were discontinued for cardiac reasons.

## Discussion

### Main findings

Our data suggest that approximately 80% of patients with suspected myocarditis did not in fact have myocarditis; only in 11% we confirmed myocarditis and 9.8% had inadequate clinical data to determine the final diagnosis.

Very few instances of myocarditis were found in patients treated in SLaM who were not receiving clozapine treatment. This represents a different population of patients, many without psychosis, not prescribed antipsychotics and living in the community, so any direct comparison is invalid. Nevertheless, our findings support the current understanding that the risk of myocarditis is greatly increased in clozapine users compared with the general population,^12^ and also compared with patients prescribed antipsychotics other than clozapine, where there were no confirmed instances.

The principal problem in identifying CIM (and myocarditis in general) is that signs and symptoms alone are of limited specificity. Some individuals may be asymptomatic, and the typical symptomatology, including lethargy, tachycardia and dizziness, is often indistinguishable from common clozapine-induced adverse effects. This often results in premature cessation of clozapine treatment, before CIM can be diagnosed. In our study, most symptoms, signs and lab results had low diagnostic value. Three measures (tachycardia, elevated CRP and raised troponin) had a 100% sensitivity for myocarditis, meaning that all instances with confirmed myocarditis showed these features. However, tachycardia was not useful as a diagnostic marker, since it had a low specificity, i.e. a very high proportion of false positives also had tachycardia. This finding is consistent with the known high prevalence of tachycardia in patients prescribed clozapine.^23^ Only elevated troponin had high specificity (89.1%). Both elevated troponin and CRP were found to be good diagnostic measures for myocarditis with a ROC AUC of 0.975 and 0.896, respectively. Based on our results, any increase in troponin should be a ‘red light’, and even non-severe rises of CRP might herald myocarditis. As expected, choosing a more extreme cut-off for troponin of twice the upper limit of normal or CRP > 100 increases specificity, at the cost of sensitivity.

An important finding in our study is that the latest instance of myocarditis was at 42 days from initiation, which correlates with earlier observations that CIM occurs early in treatment.^10^ Moreover, all identified instances were in patients either at their first introduction to clozapine, or whose previous clozapine trial was both short (<45 days) and terminated because of severe adverse events. These findings may justify a lower index of suspicion after 6 weeks of treatment.

### Interpretation of our findings

Our findings demonstrate the clinical utility of troponin levels as a diagnostic measure when CIM is suspected, particularly before clozapine cessation is considered. From a practical perspective, our data suggests that troponin levels greater than the upper limit of normal should warrant clozapine cessation until confirmatory tests such as an ECG or cMR are performed. To note, these confirmatory tests seem essential: despite relatively high specificity (89.1%), only half of the instances with troponin greater than normal were confirmed as myocarditis (positive predictive value of 50%). Therefore, even a typical event, such as high suspicion arising within the critical period of 6 weeks and accompanied by positive troponin (i.e.one that clearly warrants clozapine cessation until confirmatory test are performed), should not evolve into a final diagnosis of myocarditis without additional tests and cardiology consultation.

Interestingly, in our study, we found patients that safely continued clozapine treatment despite a diagnosis of CIM. Although continuing clozapine treatment in such circumstances cannot be supported by available evidence, our findings are consistent with previous reports that myocarditis can have a self-resolving course and this is an area that should be explored further in other data-sets.^22^ Although no definitive conclusion can be drawn, our data may suggest that some patients experience a short, self-remitting courses of myocarditis, and can continue to benefit from clozapine therapy.

Previous studies on CIM have reported that inflammatory markers are predictive of myocarditis and suggested that the prevalence of myocarditis is higher than actually proposed.^12^ In our study, patients who had myocarditis ruled out demonstrated a high prevalence of systemic signs of inflammation such as fever, malaise, tachycardia and even elevated CRP. However, despite clozapine maintenance in most, this systemic response subsided without any intervention. Overall, our data suggests that a non-specific inflammatory response is common when initiating clozapine, and that this inflammatory ‘clozapine storm’, that seems to occur within the first month of initiation, is not necessarily predictive of myocarditis.

### Inflammatory response

The mechanism whereby clozapine increases the risk of myocarditis is not fully understood, but the evidence that clozapine has immunomodulatory effects is mounting. The effects of clozapine on neutrophil counts are well documented, and there is increasing recognition of the ‘clozapine storm’ reaction comprising transient fever and cytokine elevation during the first few weeks of clozapine treatment.^24^ A recent study demonstrated that patients treated with clozapine had lower immunoglobulin levels, suggesting an immunomodulatory effect of clozapine.^25^ Thus, it appears that clozapine, for some individuals, invokes a systemic inflammatory response, that includes myocarditis-like symptoms, or even encompasses a myocardial–inflammatory response as part of the systemic inflammatory syndrome. Investigation into this ‘clozapine storm’, possible clozapine-induced systemic inflammatory response, might yield insights about how to decrease the intensity of inflammatory response and decrease incidence of myocarditis, and might even shed more light on the mechanism of therapeutic action of clozapine.

### Discontinuation

Previous studies showed that concerns about suspected myocarditis might lead to premature discontinuation of clozapine.^7^ Our data supported this observation, showing that in suspected CIM instances where myocarditis was ruled out, 18.3% nevertheless discontinued clozapine. Current literature has demonstrated that premature discontinuation of clozapine in TRS is often associated with poor clinical outcomes.^26^ Existing literature suggests that the monitoring burden of clozapine treatment is a frequent reason for early discontinuation of clozapine.^27^ This is likely compounded by a perception of clozapine as a dangerous drug with severe cardiac adverse effects, which likely explains the frequent discontinuation of clozapine after myocarditis is suspected but not confirmed. Once a suspicion of myocarditis has been raised and clozapine stopped, even if myocarditis is not confirmed, patients are unlikely to be offered clozapine again.

### Limitations

The knowledge among many clinicians that clozapine is associated with myocarditis is likely to raise their suspicion of myocarditis in a patient with non-specific symptoms if that patient is on clozapine than in a patient treated with a different antipsychotic. This is likely to have biased this study, which relies on clinician-initiated investigations, towards overestimating the incidence of myocarditis in clozapine-treated patients.

The NLP algorithm used to identify instances of myocarditis required two positive text references to myocarditis, an algorithm that might not be sensitive enough to have identified all instances of suspected myocarditis. However, as instances in which myocarditis did occur had significantly more mentions of myocarditis than those where it was ruled out (mean 10.70, s.d. = 16.77 *v.* 4.93, s.d. = 6.31, *P* < 0.001), it is likely that our NLP search strategy missed more suspected instances that would have been ruled out, than true instances of myocarditis, leading to a possible overestimation of the proportion of suspected myocarditis instances that are confirmed as true myocarditis.

The decision to classify instances as ‘confirmed myocarditis’ was based on available parameters that can be used to judge the probability for a ‘true’ myocarditis. Often, these were the parameters that led to the raised suspicion and evaluation. This might form a ‘tautological’ bias that might boost the diagnostical value of each parameter. However, as these parameters are the most commonly used, and the study examined naturalistic setting, it is not expected to invalidate the results.

When evaluating patients, the study cardiologists had to rely on data recorded in the files, which were sometimes not definitive. Although agreement was excellent, 9.8% of the instances could not be determined and were excluded from the analysis. In the extreme scenario, i.e. that all the undetermined cases were myocarditis, or all could be ruled out, the overall proportion of suspected cases that were true myocarditis would be 21.2% or 11.4%, respectively. It is likely that the true figure lies somewhere between these extremes. If we assume that the proportion of true myocarditis cases among these undetermined cases is the same as in the remainder of the sample, the proportion of suspected cases who were true myocarditis would be around 12.5%.

### Implications

Overall, our findings demonstrate that myocarditis can be ruled out in 80–90% of suspected instances, that clinical signs such as tachycardia are not useful discriminators, that elevated troponin or CRP should raise the index of suspicion, and that myocarditis is rare after the sixth week of treatment.

## Data availability

Data are owned by a third party, Maudsley Biomedical Research Centre (BRC) Clinical Records Interactive Search (CRIS) tool, which provides access to anonymised data derived from SLaM electronic medical records. These data can only be accessed by permitted individuals from within a secure firewall (i.e. the data cannot be sent elsewhere), in the same manner as the authors. For more information please contact: cris.administrator@slam.nhs.uk.
