# Clozapine-induced myocarditis: electronic health register analysis of incidence, timing, clinical markers and diagnostic accuracy – ERRATUM

**DOI:** 10.1192/bjp.2021.137

**Published:** 2021-12

**Authors:** Aviv Segev, Ehtesham Iqbal, Theresa A. McDonagh, Cecilia Casetta, Ebenezer Oloyede, Susan Piper, Carla M. Plymen, James H. MacCabe

**Keywords:** Esketamine, ketamine, depression, antidepressants, suicide, corrigendum

An incorrect version of figure 3 was published in the above article. This has now been corrected in both the online PDF and HTML versions of this article. The Publisher apologises for this error.
